# Tetraspanins: integrating cell surface receptors to functional microdomains in homeostasis and disease

**DOI:** 10.1007/s00430-020-00673-3

**Published:** 2020-04-09

**Authors:** Daniel Kummer, Tim Steinbacher, Mariel Flavia Schwietzer, Sonja Thölmann, Klaus Ebnet

**Affiliations:** 1 Institute-Associated Research Group: Cell Adhesion and Cell Polarity, Institute of Medical Biochemistry, ZMBE, University of Münster, Münster, Germany; 2 Interdisciplinary Clinical Research Center (IZKF), University of Münster, Münster, Germany; 3 Cells-In-Motion Cluster of Excellence (EXC1003-CiM), University of Münster, Münster, Germany; 4 Institute of Medical Biochemistry, ZMBE, Von-Esmarch-Str. 56, 48149 Münster, Germany

**Keywords:** Tetraspanins, Junctional adhesion molecules, Signaling, Microdomains

## Abstract

Tetraspanins comprise a family of proteins embedded in the membrane through four transmembrane domains. One of the most distinctive features of tetraspanins is their ability to interact with other proteins in the membrane using their extracellular, transmembrane and cytoplasmic domains, allowing them to incorporate several proteins into clusters called tetraspanin-enriched microdomains. The spatial proximity of signaling proteins and their regulators enables a rapid functional cross-talk between these proteins, which is required for a rapid translation of extracellular signals into intracellular signaling cascades. In this article, we highlight a few examples that illustrate how tetraspanin-mediated interactions between cell surface proteins allow their functional cross-talk to regulate intracellular signaling.

## Introduction

The translation of extracellular signaling cues into intracellular signaling cascades is frequently mediated by the assembly of signaling complexes at the plasma membrane. Different mechanisms have evolved how these signaling complexes propagate the signals into the interior of the recipient cell. Signaling by G-protein-coupled receptors (GPCRs) is mediated by conformational changes induced by ligand binding resulting in the dissociation of heterotrimeric G-proteins and activation of signaling cascades through the generation of second messengers [1]. Signaling through receptor tyrosine kinases (RTKs) generally involves receptor dimerization as a consequence of ligand binding, which results in a release of the intracellular autoinhibited kinase domain into the active configuration [2]. The consequence of *trans*-phosphorylation is strongly increased kinase activity, phosphorylation of multiple tyrosine residues, recruitment of adaptor proteins, and assembly of signaling complexes [2].

Signaling events are frequently restricted to specific subcellular sites. This site-specific localization of signaling events can be mediated by physically linking the signaling molecules to cell adhesion receptors present at specific locations, such as sites of cell–matrix adhesion like focal adhesions (FA) [3], or sites of cell–cell adhesion like adherens junctions (AJ) [4], tight junctions (TJ) [5], or synapses [6]. One mechanism underlying the assembly of larger signaling complexes at these sites is the use of scaffolding proteins which directly interact with cell adhesion receptors. These scaffolding proteins contain multiple protein–protein interaction domains such as PDZ domains, SH3 domains, or FERM domains [7], allowing them to recruit cytoplasmic signaling proteins at specific sites for functional interactions. One additional mechanism is based on lateral associations of cell adhesion receptors with other integral membrane proteins through linker proteins that are also embedded in the plasma membrane but can interact with several other integral membrane proteins simultaneously. By generating microdomains in the membrane, these lateral associations can promote the assembly of higher order protein complexes.

Tetraspanins (Tspans, also called transmembrane 4 superfamily (TM4SF) proteins), a family of integral membrane proteins consisting of 33 members in humans [8, 9], have emerged as bona fide organizers of microdomains in the plasma membrane [10–15]. Tspans contain four α-helical transmembrane (TM) regions (TM1–TM4), two extracellular (EC) domains (EC1 and EC2), and three cytoplasmic (CP) regions, (N-terminus, C-terminus and a short loop that connects TM2 and TM3) [10]. These topological domains contribute differently to the structure of tetraspanins and to their ability to undergo intramolecular and intermolecular interactions [15]. The most distinctive feature that qualifies Tspans to organize larger protein complexes in the membrane is their ability to use several of their topological regions of the protein, i.e., the EC domains, the TM domains and the CP regions for interactions with other proteins [16, 17]. Of the extracellular domains, EC2 is predominantly involved in heterophilic interactions with other proteins and mainly responsible for the specificities underling these interactions [18]. In addition, the EC2 domain can also mediate tetraspanin dimerization [19]. The TM domains undergo intramolecular interactions that support the functional protein conformation, but they can also mediate homophilic and heterophilic Tspan–Tspan interactions [15, 16, 20, 21]. The CP regions of some Tspan proteins have been found to interact with cytosolic signaling or scaffolding proteins. These include conventional PKCs, which bind to the N-terminal CP region of CD9 [22], small GTPases like Rac1 which interact with the C-terminal CP region of CD81 [23], and PDZ domain-containing scaffolding proteins like Syntenin-1 or Pick-1, which bind to PDZ domain-binding motifs at the C-termini of CD63 or Tspan7, respectively [24, 25]. The multiple possibilities to interact with other proteins enables Tspans to assemble microdomains or clusters. Tetraspanin clusters cover an area of approximately 100–400 nm^2^ [12, 15, 26, 27]. More recent studies using superresolution microscopy indicate that Tspans form nanoclusters composed of a limited number (less than 10) of selected Tspans with only minor overlaps with other Tspan nanoclusters [15]. The assembly of protein complexes to so-called Tspan-enriched microdomains (TEMs) in the plasma membrane is most liklely the principal biological function of Tspans. In this review article, we will present some examples to illustrate the molecular mechanisms through which Tspans exert their function as organizers of signaling complexes during homeostasis and disease with a particular emphasis on their function in connecting immunoglobulin superfamily (IgSF) proteins to other cell surface receptors.

## Tspans as linkers between IgSF members and integrins

### Tspans link IgSF family proteins to integrins to regulate angiogenesis

Angiogenesis describes the formation of new blood vessels from the pre-existing vasculature [28]. It is initiated by proangiogenic factors such as bFGF or VEGF which act on their receptors on endothelial cells to trigger a series of events including the polarization, proliferation and migration of endothelial cells, remodeling of cell–matrix and cell–cell adhesion, and eventually lumen formation [29]. The signaling activities of angiogenic growth factors like bFGF/FGF2 or VEGF depend in many cases on a specific cooperation with integrins [30]. For example, VEGF-regulated angiogenesis requires a functional αvβ5 integrin, whereas bFGF-regulated angiogenesis requires αvβ3 integrin [31]. The functional coupling of VEGF and bFGF signals to different integrins results in different signaling outcomes [32, 33], suggesting that the physical connection of growth factor receptors or cell adhesion receptors to specific integrins dictates signaling pathways. Recent evidence has identified tetraspanins as linkers between growth factor receptors or adhesion receptors and integrins.

#### Tspan CD63 links VEGFR2 to β1 integrins in endothelial cells

**Fig. 1:**
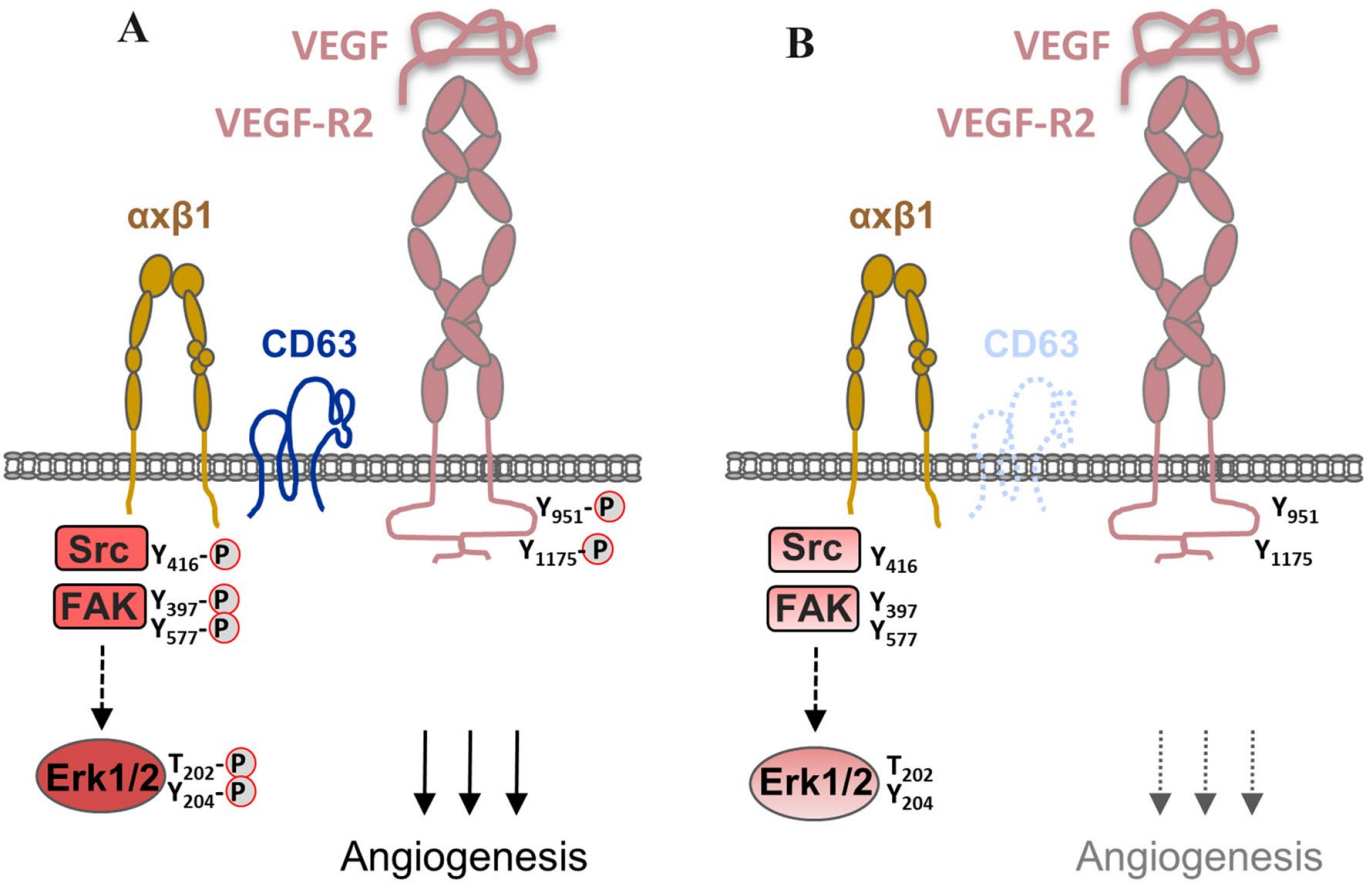
Regulation of VEGFR2 signaling in endothelial cells by tetraspanin CD63. **a** Tetraspanin CD63 links β1 integrins to VEGFR2 in endothelial cells. VEGF triggers signaling pathways downstream of β1 integrin as well as of VEGFR2. **b** In the absence of CD63, VEGF-mediated signaling is impaired. Note that the absence of CD63 not only impairs integrin-mediated signaling involving Src, FAK and Erk1/2, but also VEGFR2 phosphorylation

Tetraspanin CD63 is highly expressed in endothelial cells [34] and interacts with various integrins including β1 and β3 integrins in primary endothelial cells [35, 36]. The VEGFR2 has also been found to interact with β1 integrins which allows endothelial cells to respond to extracellular matrix-immobilized angiogenic VEGF [37]. Interestingly, CD63 interacts not only with β1 and β3 integrins but also with VEGFR2 suggesting that it serves to link VEGFR2 to the integrins [36]. Consistent with this notion, CD63 depletion diminishes the association between β1 integrin and VEGFR2. CD63 depletion also results in diminished activation of VEGFR2 and in diminished activities of key signaling components present at cell–matrix adhesions such as FAK, Src and Erk1/2 in response to VEGF [36]. These findings strongly suggest that CD63 is required to link VEGFR2 to β1 integrin, probably to establish a functional cross-talk between activated VEGFR2 and β1 integrin-associated signaling molecules (Fig. 1).

#### Tspan CD9 links JAM-A to αvβ3 integrin in endothelial cells

**Fig. 2:**
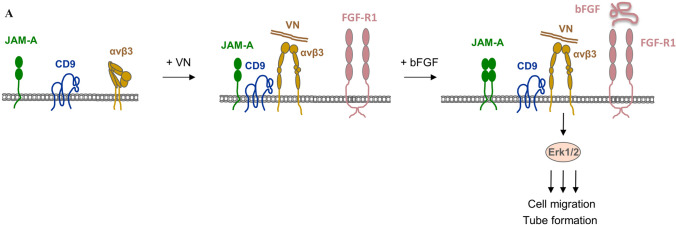
Regulation of bFGF signaling in endothelial cells by tetraspanin CD9. **a** Tetraspanin CD9 links cell adhesion molecule JAM-A to αvβ3 integrin to generate a ternary JAM-A–CD9–αvβ3 integrin complex. Left panel: Under resting conditions JAM-A, CD9 and αvβ3 integrin are weakly associated. Middle panel: Integrin activation by vitonectin (VN) increases complex formation, probably by promoting lateral association between activated αvβ3 integrin and CD9. Right panel: bFGF stimulation triggers JAM-A release from the complex concomitant with Erk1/2 activation

Similar to CD63, tetraspanin CD9 interacts with various integrins including a number of β1 and β3 integrins, but at the same time with a number of IgSF members [12]. CD9 has also been shown to regulate ERK signaling by modulating ADAM17 protease activity, thereby affecting cellular and pathophysiological processes such as cell migration, wound healing and virus infection [38, 39] (Mikulicic et al. this issue). More recently, CD9 has been identified as an interaction partner of JAM-A [40], a cell adhesion receptor of the IgSF that is expressed by different cell types including endothelial cells [41]. CD9 links JAM-A to αvβ3 integrin to form a ternary JAM-A–CD9–αvβ3 integrin complex. Importantly, the depletion of either JAM-A or CD9 impairs bFGF-triggered MAPK activation but has no effect on VEGF-triggered MAPK activation [40], which is in line with previous findings that bFGF cooperates with αvβ3 integrin in angiogenic signaling [31]. CD9 thus promotes the formation of a bFGF-specific signaling unit in endothelial cells by linking the cell adhesion receptor JAM-A to αvβ3 integrin. How the bFGF-triggered signal is propagated is less clear. Integrin activation promotes ternay complex formation suggesting that the open conformation of αvβ3 integrin more readily interacts with CD9. In contrast, bFGF induces the release of JAM-A from the ternary complex [40, 42] (Fig. 2). This latter observation has been interpreted in a way that preferentially monomeric JAM-A might be incorporated in the complex and that its release induced by bFGF might allow the formation of a signaling-competent dimer [40].

### A role for CD9 in linking IgSF proteins to integrins in platelets?

Tetraspanin CD9 is also expressed on platelets in which it is the most abundant tetraspanin localized at the surface [43]. Among other partners, it interacts with αIIbβ3 integrin [44], which is a receptor for fibrinogen and the dominant integrin on platelets [45]. Interestingly, JAM-A has been demonstrated to interact with both CD9 and with αIIbβ3 integrin in platelets [46], which suggests that similar to endothelial cells, a ternary JAM-A–CD9–β3 integrin complex might exist in platelets. Functional analyses further support the existence of such a ternary complex in platelets. Interestingly, however, this complex seems to play an inhibitory role in platelet activation, which contrasts to its activating function in endothelial cells.

**Fig. 3:**
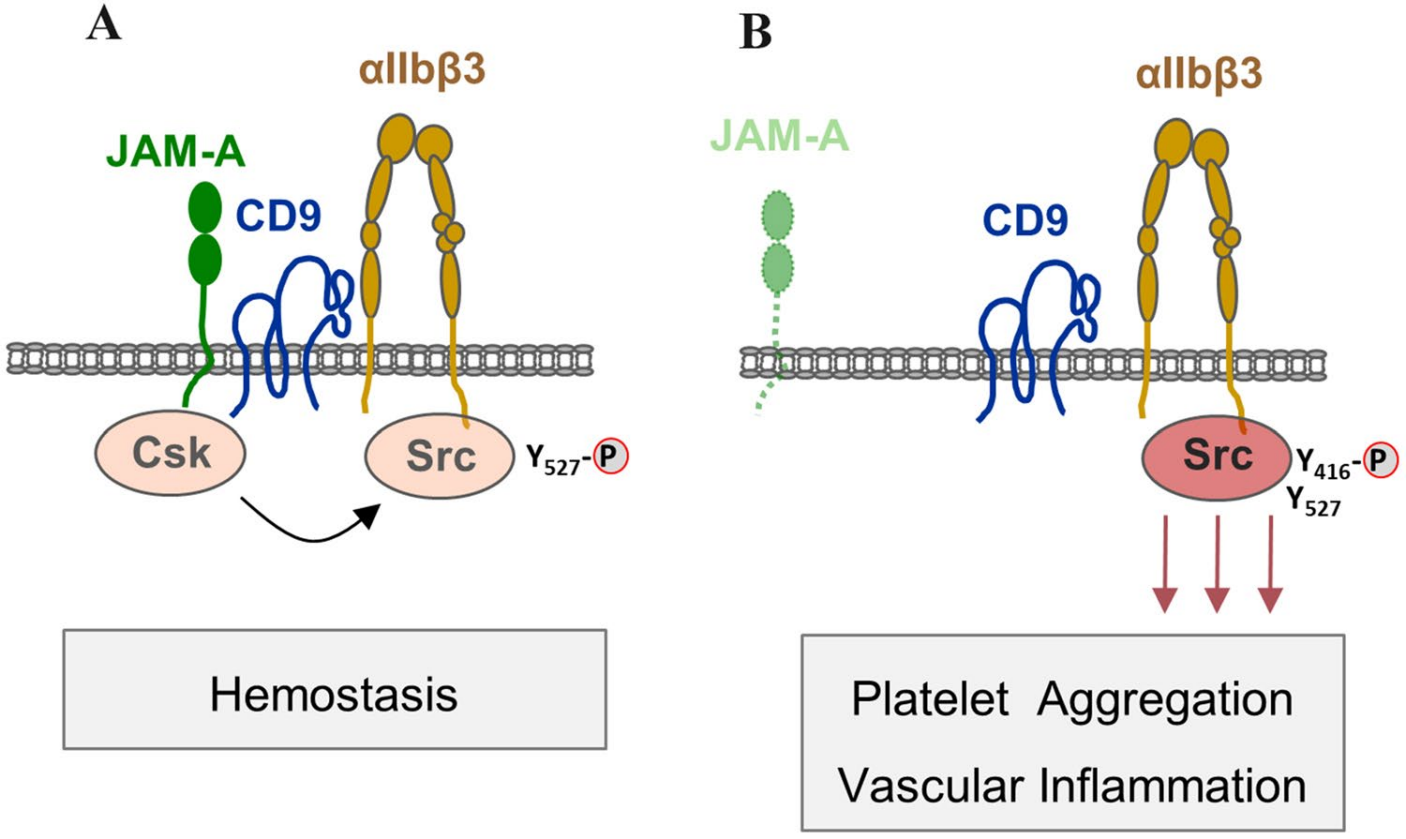
Regulation of Src activity in platelets by tetraspanins CD9? **a** Tetraspanin CD9 interacts with both cell adhesion molecule JAM-A and αIIbβ3 integrin in platelets. CD9 possibly serves to link JAM-A to αIIbβ3 integrin to enable functional interaction between JAM-A-associated Csk and αIIbβ3 integrin-associated Src kinase. **b** In the absence of JAM-A, functional interaction of Csk and Src is prevented and Src becomes active, which leads to uncontrolled stimulation of Src-dependent signaling pathways. As a consequence, platelets become hyperreactive leading to thrombus formation and vascular inflammation. Similar to endothelial cells, CD9 interacts with both JAM-A and the β3 integrin. Note that its function as linker between JAM-A and αIIbβ3 integrin has not been proven, yet

In the absence of CD9, the binding of fibrinogen to stimulated platelets and the inclination to thrombus formation are significantly enhanced, suggesting that CD9 inhibits αIIbβ3 integrin-mediated signaling [47]. Similarly, in the absence of JAM-A, platelets are hyperreactive and release significantly more pro-inflammatory chemokines, which is accomapgnied by increased thrombus formation and vascular inflammation [48, 49]. This inhibitory activity of JAM-A is mediated by the recruitment of the Src-inhibitory kinase Csk to αIIbβ3 integrin-associated Src [42]. Given that CD9 and JAM-A have in common an inhibitory function to platelet activation, and that both exist in a complex with αIIbβ3 integrin, it is tempting to speculate that CD9 links JAM-A to αIIbβ3 integrin to allow a functional interaction of JAM-A-associated Csk with αIIbβ3 integrin-associated Src. Thus, CD9 would have the same function in two different cell types, i.e., connecting the IgSF member JAM-A to a β3 integrin (αvβ3 in endothelial cells, αIIbβ3 integrin in platelets), with, however, different signaling outcomes in the two cell types (Fig. 3).

### Tspans link EWI family proteins to integrins to regulate integrin-dependent functions in tumor cells

Tetraspanins CD9 and CD81 interact with members of a small subfamily of the IgSF of proteins, which is characterized by a conserved Glu-Trp-Ile (EWI) motif. Two members of this family, i.e., EWI motif-containing protein 2 (EWI-2) and EWI-F, have been identified as tetraspanin partners on the basis of their interaction with CD9 and CD81 [50–52]. EWI-2 contains four Ig-like domains, EWI-F contains six Ig-like domains.

The association of CD9 and CD81 with EWI-2 has been characterized in more detail. The interaction of EWI-2 with CD9 and CD81 is most likely direct and mediated by the extracellular domains of the proteins with contribution of the transmembrane domain and Cys residues in the cytoplasmic domain of EWI-2 that are palmitoylated [50, 52, 53]. EWI-2 not only interacts with CD9 and CD81 but also with α3β1 integrin, which is a receptor for laminin-5, and both CD9 and CD81 can link EWI-2 to α3β1 integrin [54]. Ectopic expression of EWI-2 impairs the motility of cells on laminin-5, suggesting that EWI-2 influences α3β1 integrin-dependent functions [54]. These findings thus indicate that CD9 and CD81 link EWI-2 to a specific integrin, i.e., α3β1 integrin, to regulate cellular functions that depend on this particular integrin, such as cell migration or scattering of carcinoma cells [55]. The molecular mechanism through which EWI-2 influences these functions are still unexplored.

## EWI family proteins regulate Tspan interactions with TGFβ receptors to regulate receptor signaling

The regulation of EWI protein interactions with other integral membrane proteins by tetraspanins can be mutual and even occur in a negative manner. As opposed to the recruitment of EWI-2 to α3β1 integrin by CD9 and CD81 to allow functional interaction between EWI-2 and the integrin, the recruitment of CD9 and CD81 to TGFβ receptors is prevented by EWI-2 to limit TGFβ signaling [56].

TGFβ signaling plays a central role during epithelial-to-mesenchymal transition (EMT), a process that is critical during development but when inadvertently reactivated in the adult organism contributes to tumor progression by facilitating invasion and metastasis [57]. TGFβ signals by inducing hetero-dimerization of two receptors, the Ser/Thr kinases TGFβ receptor I (TβRI) and TβRII, resulting in the activation of SMAD proteins, a family of cytoplasmic proteins that form transcriptional complexes which interact with other transcription factors to regulate a plethora of genes [58]. In melanoma cells, TGFβ signaling contributes to the development of an EMT-like phenotype and to mestasasis [59]. Intriguingly, TGFβ signaling has different outcomes in melanocytes versus melanoma cells. It has cytostatic effects in melanocytes but promotes proliferation and survival in melanoma cells [60].

**Fig. 4:**
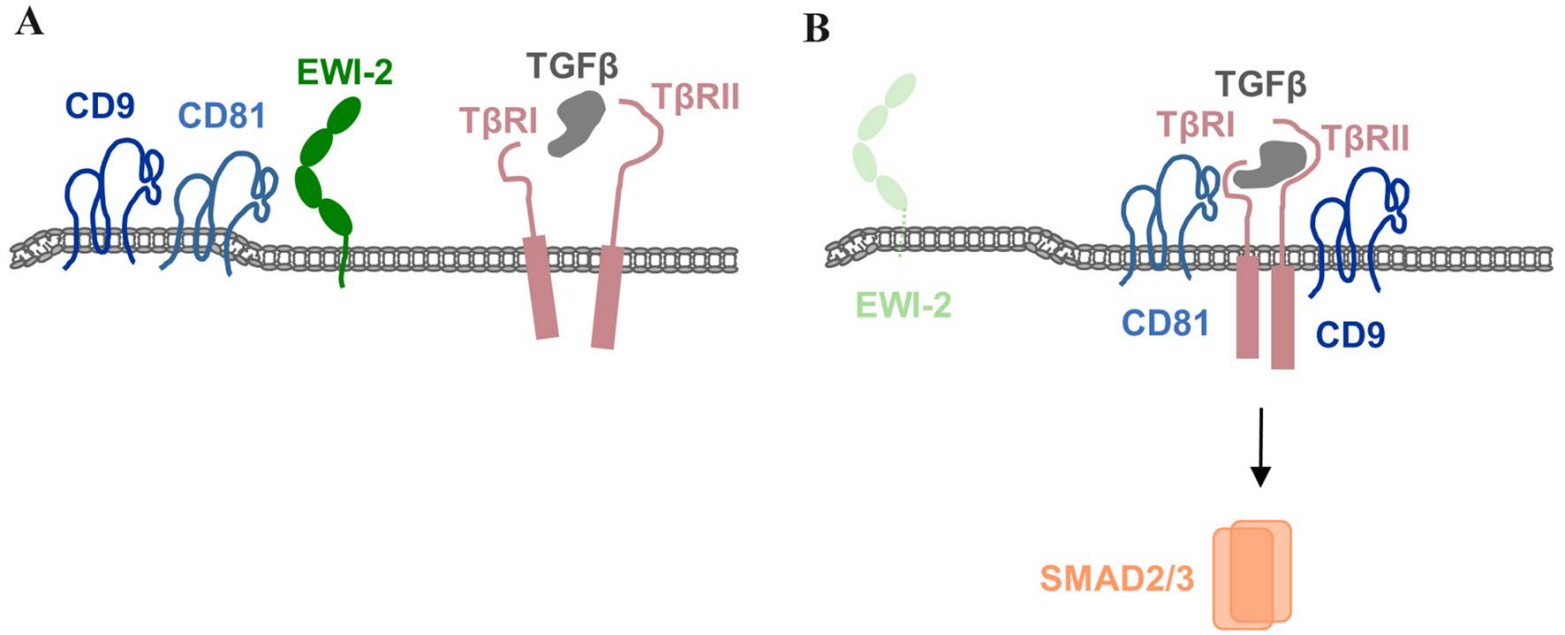
Regulation of TGFβ receptor signaling in melanoma cells by tetraspanins CD9 and CD81 and their partner EWI-2. **a** In early stage, melanoma cells EWI-2 is expressed and interacts with CD9 and CD81. CD9 and CD81 are sequestered from the TGFβ receptor thereby inhibiting the formation of a functional heterodimeric TβRI–TβRII complex. **b** In later stage melanoma cells, EWI-2 is downregulated allowing CD9 and CD81 to interact with TβRII thereby promoting the formation of a signaling-competent TGFβ receptor. The activation of Smad proteins promotes invasion and metastasis

A recent study indicates that the tetraspanin partner proteins EWI-2 negatively regulates TGFβ signaling, and that EWI-2 regulates TGFβ signaling activity through CD9 and CD81 [56]. In early stage melanoma cells, when TGFβ signaling is low, EWI-2 is strongly expressed and interacts with CD9 and CD81, thereby sequestering CD9 and CD81 from the TGFβ receptor complex. However, in later stage melanoma cells when TGFβ signaling is high, EWI-2 is downregulated, which allows its two tetraspanin partners CD9 and CD81 to engage with TGFβ receptors and stabilize a signaling active TGFβ receptor complex resulting in increased Smad signaling [56] (Fig. 4). TGFβ receptor signaling thus provides a scenario in which tetraspanins are sequestered from a signaling receptor in the plasma membrane by interacting with a tetraspanin binding partner.

## Tspans organize nanoclusters of co-receptors to regulate B cell activation upon antigen recognition

The B cell receptor (BCR) mediates the antigen-specific activation of B-lymphocytes leading to the production of antibodies. The BCR consists of membrane-bound immunoglobulins (Ig) which bind the antigens, and of the non-covalently bound co-receptors CD79A and CD79B (Igα and Igβ, respectively) which form a heterodimer that initiates intracellular signaling [61]. The intracellular signaling is initiated by phosphorylation of ITAM motifs present in the cytoplasmic domains of CD79A and CD79B by Src family kinases (SFK), which leads to an increased association of these kinases with the co-receptors and enhancement of their kinase activity. These initial signals are then propagated by the recruitment of adaptor proteins which recruit additional kinases, adaptors and phospholipases [61].

In the absence of antigens, the BCR is not silent but generates a low strength signal that is required for the survival of mature B cells [62]. Antigen binding amplifies the tonic low strength signal into a strong signal, suggesting the presence of accessory molecules which serve to increase the signaling capacity of the BCR. One of these accessory molecules is CD19, a single-pass transmembrane protein with two Ig-like domains [63]. CD19 is associated with the BCR and enhances BCR signaling, which is most likely based on its ability to serve as a substrate for SFK and at the same time as a SFK scaffold that promotes SFK activity, and in addition on its ability to regulate PI(3)K signaling [64, 65]. Interestingly, CD19 interacts with tetraspanin CD81 [66, 67]. The interaction with CD81 is necessary for CD19 surface localization [68] but also for the activation of B cells through the BCR [69]. Consistent with this function, the absence of CD81 results in a failure in mounting a humoral immune response [70, 71].

**Fig. 5:**
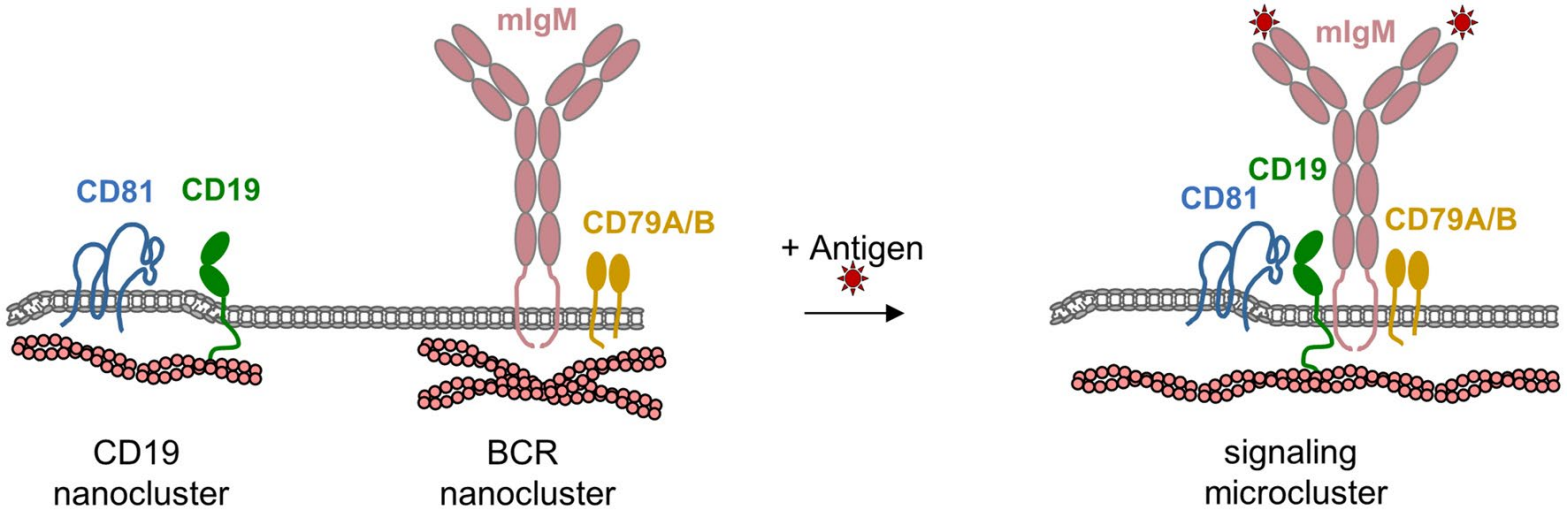
Regulation of antigen receptor signaling in B cells by tetraspanin CD81. In the absence of antigens, the BCR is organized in nanoclusters regulated by the actin cytoskeleton (left panel). Under these conditions, the BCR provides a tonic, low strength signal required for B cell survival. Binding of antigens to IgM (right panel) triggers cytoskeletal reorganization that increases the diffusibility of BCR-containing nanoclusters allowing them to interact with CD19 in CD81-containing nanoclusters, which is necessary for B cell activation and development of effector functions. *mIgM* membrane-bound IgM

More recent evidence provided a deeper insight into the role of CD81 in regulating BCR signaling through CD19. In resting B cells, IgM- and IgD-based BCRs as well as CD19 are compartimentalized in nanoclusters at the B cell surface. Disrupting the actin cytoskeleton results in increased diffusion of IgM and IgD nanoclusters triggering cytoskeleton-dependent BCR signaling. This observation is interpreted in a way that the BCR is kept immobile in a nanocluster and that its diffusibility is required for efficient BCR signaling. Of note, CD19 is organized in a separate nanocluster, and its diffusibility is not dependent on the actin cytoskeleton but on its association with CD81 [69]. In the absence of CD81, CD19 diffusibility is altered, resulting in impaired B cell activation. CD81 thus serves to immobilize CD19 in a nanocluster separate from the BCR nanoclusters. Antigen binding to the BCR increases BCR diffusion allowing functional cross-talk with CD19-containing CD81-based nanoclusters, which triggers signal amplification [69]. These findings thus highlight a function of tetraspanins in separating co-receptors such as CD19 from primary receptors (i.e., BCRs) to maintain low tonic signaling activity but allow a strong signal amplification by the rapid coalescence of preformed nanoclusters of receptors and their co-receptors (Fig. 5).

## Concluding remarks

One of the most distinctive features of tetraspanins is their ability to associate with other proteins using all of their topological doamins, i.e., their extracellular domains, transmembrane domains and cytoplasmic domains. Together with their ability to undergo homophilic and heterophilic interactions in cis with other tetraspanins, this ability allows tetraspanins to assemble large protein complexes into microdomains. Not surprisingly, tetraspanin-enriched microdomains localized at the plasma membrane are frequently involved in intercellular communication, which requires a rapid assembly of signaling complexes in response to an extracellular signal at a specific subcellular site. Rapid signaling requires close spatial proximity of primary signaling factors, such as growth factor receptors or adhesion receptor-associated signaling molecules, with their regulators. Tetraspanins are ideally suited to assemble protein complexes in which signaling molecules and their regulators are spatially segregated to prevent activation in the absence of agonist but on the other hand are close enough to allow rapid cross-talk in the presence of agonists. Recent observations in other protein-enriched signaling units, i.e., integrin-based adhesion complexes, indicate that signal propagation occurs in the absence of changes in the composition of the adhesion complex, but is regulated by the relay of phosphotyrosine-dependent signals [72]. As outlined in this review article, tetraspanins can regulate proximity as well as spatial separation of functionally interacting proteins and thus provide the structural framework for rapid regulation of signaling processes. The application of high-resolution imaging techniques that allow single protein tracking will be key to a deeper understanding of the molecular mechanism underlying the function of tetraspanins in development, homeostasis and pathophysiology.

## Abbreviations

BCR: B cell receptor
IgSF: Immunoglobulin superfamily
JAM-A: Junctional adhesion molecule-A
TEM: Tetraspanin-enriched microdomain
TM4SF: Transmembrane 4 superfamily
